# Involvement of the chemokine CCL3 and the purinoceptor P2X7 in the spinal cord in paclitaxel-induced mechanical allodynia

**DOI:** 10.1186/1744-8069-10-53

**Published:** 2014-08-15

**Authors:** Ryutaro Ochi-ishi, Kenichiro Nagata, Tomoyuki Inoue, Hidetoshi Tozaki-Saitoh, Makoto Tsuda, Kazuhide Inoue

**Affiliations:** 1 Department of Molecular and System Pharmacology, Graduate School of Pharmaceutical Sciences, Kyushu University, 3-1-1 MaidashiHigashi-ku, Fukuoka 812-8582, Japan

**Keywords:** Paclitaxel, Microglia, Chemokines, Spinal cord, Allodynia, Rats

## Abstract

**Background:**

Paclitaxel is an effective chemotherapeutic agent widely used for the treatment of solid tumors. The major dose-limiting toxicity of paclitaxel is peripheral neuropathy. The mechanisms underlying the development and maintenance of paclitaxel-induced peripheral neuropathy are still unclear, and there are no currently established effective treatments. Accumulating evidence in models of neuropathic pain in which peripheral nerves are lesioned has implicated spinal microglia and chemokines in pain hypersensitivity, but little is know about their roles in chemotherapy-induced peripheral neuropathy. In the present study, we investigated the role of CC-chemokine ligand 3 (CCL3) in the spinal cord in the development and maintenance of mechanical allodynia using a rat model of paclitaxel-induced neuropathy.

**Findings:**

Repeated intravenous administration of paclitaxel induced a marked decrease in paw withdrawal threshold in response to mechanical stimulation (mechanical allodynia). In these rats, the number of microglia in the spinal dorsal horn (SDH) was significantly increased. Paclitaxel-treated rats showed a significant increase in the expression of mRNAs for CCL3 and its receptor CCR5 in the SDH. Intrathecal administration of a CCL3-neutralizing antibody not only attenuated the development of paclitaxel-induced mechanical allodynia but also reversed its maintenance. Paclitaxel also upregulated expression of purinoceptor P2X7 receptors (P2X7Rs), which have been implicated in the release of CCL3 from microglia, in the SDH. The selective P2X7R antagonist A438079 had preventive and reversal effects on paclitaxel-induced allodynia.

**Conclusions:**

Our findings suggest a contribution of CCL3 and P2X7Rs in the SDH to paclitaxel-induced allodynia and may provide new therapeutic targets for paclitaxel-induced painful neuropathy.

## Findings

### Introduction

Paclitaxel is an effective chemotherapeutic agent widely used for the treatment of breast, ovarian, and non-small-cell lung cancer [1]. Paclitaxel often induces peripheral neuropathy, which is characterized by a sensory abnormality of extremities usually occurring in a stocking-and-glove distribution in addition to motor dysfunction in patients [2]. Peripheral neuropathy is the major dose-limiting toxicity of paclitaxel and may persist for months to years [3], having a long-term negative impact on patients’ quality of life. However, the mechanisms underlying paclitaxel-induced peripheral neuropathy remain unknown, and there are no established treatments.

A growing body of evidence indicates that activated microglia in the spinal dorsal horn (SDH) play important roles in pathological chronic pain in different animal models [4-6]. Activated microglia produce many types of inflammatory mediators, such as proinflammatory cytokines and chemokines, which contribute to the initiation and maintenance of pain hypersensitivity [6-8]. It was recently reported that paclitaxel treatment induces activation of microglia in the SDH in rats and mice [9] and that intrathecal administration of minocycline, a reagent that can inhibit microglial activation attenuates paclitaxel-induced pain hypersensitivity [10]. We have previously shown that microglia produce and release CC-chemokine ligand 3 (CCL3; also known as MIP-1α) [11] and that in a model of spinal nerve injury, spinal CCL3 expression is upregulated in microglia and contributes to mechanical allodynia [12]. Furthermore, intrathecal administration of CCL3 to naïve animals produces mechanical allodynia [12,13]. CCL3 is known to activate its cognate receptors CCR5 and CCR1 [14], and a pharmacological blockade of spinal CCL3 signaling by intrathecal administration of CCR5 antagonists reduces pain hypersensitivity after traumatic nerve injury [12,15]. These results suggest that spinal CCL3 plays an important role in traumatic nerve injury-induced allodynia. However, there are no reports demonstrating whether CCL3 in the spinal cord contributes to chemothe rapy-induced neuropathic pain. Thus, the aim of the present study was to investigate the role of spinal CCL3 in mechanical allodynia using a rat model of paclitaxel-induced neuropathy.

## Methods

All experimental procedures were approved by the Institutional Animal Care and Use committee review panels at Kyushu University.

Male Sprague–Dawley rats (8–11 weeks old) were obtained from Japan SLC (Hamamatsu, Japan). The rats were housed at a temperature of 22 ± 1°C with a 12-hour light–dark cycle and had *ad libitum* access to food and water.

Paclitaxel (LKT Laboratories, St. Paul, USA) was dissolved in a 1:1 mixture of ethanol and Cremophor EL (Sigma-Aldrich, St. Louis, USA) to make a stock solution of 12 mg/mL. Prior to administration, the paclitaxel solution was further diluted with sterile saline (1:3). Under isoflurane (2%) anesthesia, rats were administered the solution via the tail vain on days 0 and 3 after paw withdrawal threshold was measured. We used a previously characterized model of paclitaxel-induced peripheral neuropathy produced by repeated infusions of paclitaxel at a cumulative dose of 36 mg/kg (2 × 18 mg/kg, 3 days apart) [9]. Control rats received equivalent volumes of the Cremophor/ethanol vehicle.

For immunohistochemical experiments, rats were deeply anesthetized by pentobarbital and perfused transcardially with phosphate-buffered saline (PBS, composition in mM: NaCl 137, KCl 2.7, KH_2_PO_4_ 1.5, NaH_2_PO_4_ 8.1; pH 7.4) followed by ice-cold 4% paraformaldehyde/PBS. The L5 segment of the lumbar spinal cord was removed, postfixed in the same fixative, and placed in 30% sucrose solution for 24 hr at 4°C. Transverse L5 spinal cord sections (30 μm) were cut on a Leica CM 1850 cryostat (Leica Biosystems, Wetzlar, Germany) and incubated for 2 hr at room temperature in a blocking solution (3% normal goat serum), and then incubated for 48 hr at 4°C in the primary antibody for ionized calcium-binding adapter molecule 1 (Iba1, 1:2000, Wako, Osaka, Japan), a marker of microglia. Spinal sections were incubated with secondary antibodies conjugated to Alexa Fluor 488 (1:1000, Life Technologies Japan, Tokyo, Japan) and mounted in Vectashield containing 4',6-diamidino-2-phenylindole (DAPI, Vector Laboratories, Burlingame, USA). Two to three sections from the L5 spinal cord segments of each rat were randomly selected and analyzed using an LSM510 Imaging System (Carl Zeiss Japan, Tokyo, Japan). The numbers of Iba1^+^ cells in the SDH (lamina I – IV) were counted.

For quantitative real-time PCR, rats were deeply anesthetized with pentobarbital, perfused transcardially with PBS, and the L5 spinal cord was removed immediately. The tissues were separated into ventral and dorsal horn. The sample was homogenized with TRIsure (Bioline, London, UK) and RNA was purified using an RNeasy mini plus kit (Qiagen, Valencia, USA). The amount of RNA was quantified using NanoDrop spectrophotometer (Thermo Scientific, Wilmington, USA). RNA was transcribed using PrimeScript Reverse Transriptase (Takara Bio, Otsu, Japan). Quantitative PCR was performed using Premix Ex *Taq* (Takara) together with a 7500 real-time PCR system (Life Technologies Japan, Tokyo, Japan), and the data were analyzed using 7500 System SDS Software 1.3.1 (Life Technologies Japan, Tokyo, Japan). Expression levels of genes of interest were normalized to the values for glyceraldehyde-3-phosphate dehydrogenase (GAPDH) and were expressed as fold change over control rats. The sequences of TaqMan primer pairs and probes are described below: rat Iba1, 5′-GATTTGCAGGGAGGAAAAGCT-3′ (forward), 5′-AACCCCAAGTTTCTCCAGCAT-3′ (reverse), 5′-CAGGAAGAGAGGTTGGATGGGATCAA-3′ (Taqman probe); rat CCL3, 5′-CCACTGCCCTTGCTGTTCTT-3′ (forward), 5′-GCAAAGGCTGCTGGTTTCAA-3′ (reverse), 5′-CGCCATATGGAGCTGACACCCCG-3′ (Taqman probe); rat CCR1, 5′-CTAAGATGGCTAGGGCCCAAATA-3′ (forward), 5′-TCCCTGAGGGCCCGAACTGTCA-3′ (reverse), 5′-CCTGGGCTTATACAGTGAGATCTTC-3′ (Taqman probe); rat CCR5, 5′-GACCGGGTATAGACTGAGCTTACAC-3′ (forward), 5′-ACTCTTGGGATGACACACTGCTGCCTC-3′ (reverse), 5′-CAGGCAATGCAGGTGACAGA-3′ (Taqman probe); and rat purinoceptor P2XR7, 5′-CATGGAAAAGCGGACATTGA-3′ (forward), 5′-CCAGTGCCAAAAACCAGGAT-3′ (reverse), 5′-AAAGCCTTCGGCGTGCGTTTTGA-3′ (Taqman probe).

Mechanical allodynia was assessed using von Frey filaments (North Coast Medical, Gilroy, USA). Rats were placed in an aluminum cage with a wire mesh grid floor in a quiet room, 30 min before the start of testing. The von Frey filament (1.0–15.0 g) was inserted through the mesh floor bottom and was applied to the middle of the plantar surface of the hindpaw. The 50% paw withdrawal threshold (PWT) was determined using the up-down method [16].

For intrathecal administration, under isoflurane (2%) anesthesia, rats were implanted with a 32-gauge intrathecal catheter (ReCathCo, Allison Park, USA) through the atlanto-occipital region into the lumbar enlargement of the spinal cord. Seven days after implantation, the catheter placement was verified by the observation of transient hindpaw paralysis induced by intrathecal injection of lidocaine (2%, 5 μL). Animals that failed to display paralysis following lidocaine administration were not included in the experiments. To test for possible effects on the development of paclitaxel-induced mechanical allodynia, rats were injected with a CCL3-neutralizing antibody (4 ng/10 μL; R&D Systems, Minneapolis, UAS) (or control IgG2A) and the selective P2X7R antagonist A438079 (1 μg/10 μL, Tocris Bioscience, Bristol, UK) (or PBS) once a day for 7 days starting from 1 day before the first injection of paclitaxel. Intrathecal administration of these drugs was done soon after the completion of behavioral measurement (10:00 ~ 12:00). For experiments testing their effects on maintenance of paclitaxel-induced mechanical allodynia, these agents were administered intrathecally to paclitaxel-treated rats, once, on day 7.

Data are expressed as the means ± SEM. Statistical analyses of the results were conducted with the Student’s t test, one-way ANOVA with post hoc Dunnett’s multiple comparisons, and two-way ANOVA with Bonferroni’s post hoc analysis. The threshold for statistical significance was set at a *P* value < 0.05.

## Results

**Figure 1:**
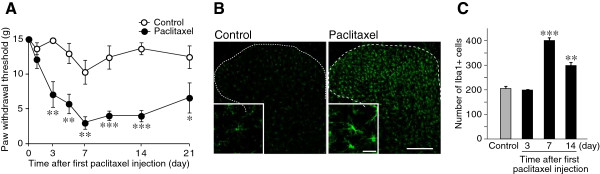
**Administration of paclitaxel causes activation of microglia in the SDH. (A)** Threshold for withdrawal in response to mechanical stimulation to the hindpaw before and after injection of vehicle or paclitaxel (**P < 0.01, *** *P* < 0.001 vs. vehicle group, *n* = 6). **(B)** Immunofluorescence for Iba1, a marker of microglia, in the SDH of vehicle (control)- or paclitaxel-treated rats (Scale bar, 200 μm). Insets: high magnification images (Scale bar, 20 μm). **(C)** The number of microglia labeled with Iba1 per 6 × 10^5^ μm in the SDH of paclitaxel-treated rats (n = 3, **P < 0.01, ***P < 0.001, vs. naïve control rats). Values are means ± s.e.m.

First, to confirm mechanical pain hypersensitivity in rats that had been administered paclitaxel via the dosing regimen used in this study, we measured PWT in response to mechanical stimulation of paclitaxel-treated rats. As shown in Figure 1A, paclitaxel produced a profound, long-term decrease in PWT. We next immunohistochemically examined activation of microglia in the SDH using an antibody against Iba1, a marker of microglia, in paclitaxel-treated rats. In the L5 SDH of paclitaxel-treated rats, Iba1 immunofluorescence was clearly increased (Figure 1B). At high magnification, Iba1-positive microglia showed an activated morphology such as thickened cell bodies and retracted processes (Figure 1B insets). The number of Iba1-positive microglia in the L5 SDH was significantly increased on days 7 and 14 after the first injection of paclitaxel (Figure 1C). In addition, we also observed a significant increase in the level of mRNA for Iba1 in the SDH (data not shown). These results together indicate that microglia become activated in the SDH of paclitaxel-treated rats.

**Figure 2:**
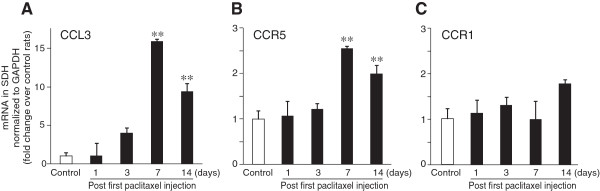
**Upregulation of CCL3 and CCR5 mRNAs in the SDH following administration of paclitaxel.** Real-time RT-PCR analyses of mRNA levels for CCL3 **(A)**, and the chemokine receptors CCR1 **(B)** and CCR5 **(C)** in the SDH of paclitaxel- and vehicle-treated rats at each time point after the first injection of paclitaxel (n = 4–6, **P < 0.001 vs. control). Values represent the relative ratio of each mRNA (normalized to the level of GAPDH mRNA) in paclitaxel-treated rats to that in naїve control rats (fold change over control rats). Values are means ± s.e.m.

**Figure 3:**
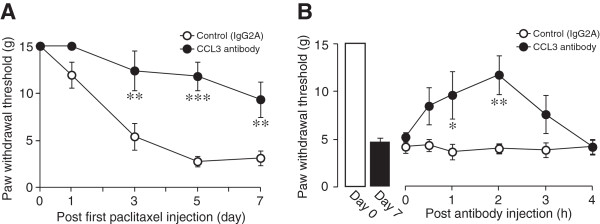
**A CCL3-neutralizing antibody suppresses the development and maintenance of paclitaxel-induced mechanical allodynia.** Intrathecal administration of a CCL3-neutralizing antibody (4 ng/10 μL) or control IgG2A was done once a day for 7 days (from a day before the first paclitaxel injection until day 6) **(A)** or once on day 7 after the first paclitaxel injection **(B)** (n = 6, *P < 0.05, **P < 0.01, ***P < 0.001, vs. control group). Values are means ± s.e.m.

To examine changes in expression of CCL3 in the SDH, we performed real-time RT-PCR analysis. Seven days after the first paclitaxel injection, the expression of CCL3 mRNA was markedly increased in the SDH (Figure 2A). The increase in CCL3 mRNA in the SDH was detected as early as day 3 (but was not statistically significant at this time), and the highest level was observed on day 7. The upregulation of CCL3 was still evident, to a slightly lesser extent, on day 14 (Figure 2A). The time course of CCL3 upregulation paralleled that of the number of microglia in the SDH (Figure 1C). We also examined the expression of CCR5 and CCR1 (receptors for CCL3 [14]) mRNAs in the SDH. CCR5 expression was significantly increased in the SDH on days 7 and 14 after the first paclitaxel administration (Figure 2B). By contrast, the CCR1 mRNA expression level was unchanged at all time points tested (Figure 2C).To determine the role of spinal CCL3 in paclitaxel-induced mechanical allodynia, we tested the effect of a CCL3-neutralizing antibody on the PWT in paclitaxel-treated rats. Repeated intrathecal injection of an anti-CCL3 antibody [once a day from day 0 (before the first paclitaxel injection) until day 6] prevented the decrease in PWT in paclitaxel-treated rats (Figure 3A). We also tested the effect of a single intrathecal injection of the CCL3 antibody on day 7 after the first paclitaxel injection, a time corresponding to the highest expression level of CCL3. Intrathecal CCL3 antibody injection significantly increased the PWT in paclitaxel-treated rats (Figure 3B). These results indicate that spinal CCL3 contributes to the development and maintenance of paclitaxel-induced mechanical allodynia.

**Figure 4:**
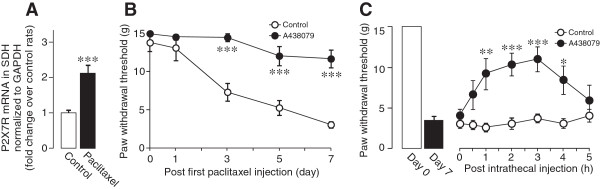
**P2X7Rs are upregulated by paclitaxel treatment and contribute to mechanical allodynia. (A)** Real-time RT-PCR analysis of P2X7R mRNA in the SDH of paclitaxel- and vehicle-treated rats 7 days after the first injection of paclitaxel (n = 6, ***P < 0.001). Values represent the relative ratio of P2X7R mRNA (normalized to the level of GAPDH mRNA) in paclitaxel-treated rats to that in vehicle control rats (fold change over control rats). **(B)** A438079 (a selective P2X7R antagonist; 1 μmol/10 μL) was intrathecally administered once a day for 7 days (from a day before the first paclitaxel injection to day 7) (n = 6, ***P < 0.001, vs. vehicle-treated control group). **(C)** A438079 (1000 nmol/10 μL) was intrathecally administered once on day 7 (n = 6, *P < 0.05, **P < 0.01, ***P < 0.001 vs. vehicle-treated control group). Values are means ± s.e.m.

We have previously demonstrated that activating purinergic P2X7Rs in cultured microglia causes release of CCL3. Thus, we further investigated the involvement of spinal P2X7Rs. We examined expression of P2X7Rs in the SDH and found that paclitaxel treatment significantly increased expression of P2X7R mRNA in the SDH on day 7. To determine the role of spinal P2X7Rs in the development of mechanical allodynia, we repeatedly administered A438079, a selective antagonist for P2X7Rs [17,18], into the intrathecal space. As shown in Figure 4B, repeated intrathecal administration of A438079 prevented the decrease in the PWT in paclitaxel-treated rats (Figure 4B). We also found that a single intrathecal administration of A438079 to paclitaxel-treated rats on day 7 reversed mechanical allodynia (Figure 4C).

## Discussion

In the present study, we demonstrated for the first time that the chemokine CCL3 in the spinal cord is involved in the mechanical allodynia caused by paclitaxel treatment in rats. Our behavioral data showing both the preventive and reversal effects of chronic and single intrathecal administration, respectively, of a CCL3-neutralizing antibody on the paclitaxel-induced allodynia suggest that CCL3 plays important roles not only in the development of paclitaxel-induced allodynia, but also in its maintenance. Although the type of cells responsible for upregulating CCL3 expression in the SDH of paclitaxel-treated rats remains to be determined, the temporal correlation between the upregulation of CCL3 expression and the increase in the number of microglial cells in the SDH leads us to hypothesize that the CCL3 may be derived from activated microglia. Indeed, the morphological hypertrophy of microglia and the increase in the number of microglia, both of which are major immunohistochemical features of microglial activation, were evident in the SDH of paclitaxel-treated rats. Furthermore, our previous study revealed production and release of CCL3 from cultured microglia [11]. In a recent study using rat brain slices *in vitro*, neuronal injury was shown to induce the microglial production of CCL3 [19]. We have also recently demonstrated upregulation of CCL3 expression in spinal microglia after traumatic nerve injury in rats [12], which strongly supports our hypothesis. Moreover, P2X7Rs have been implicated in CCL3 release from microglia [11], and our study showed that pharmacological blockade of spinal P2X7Rs suppressed paclitaxel-induced mechanical allodynia, the effect of which was similar to that of the CCL3-neutralizing antibody. P2X7R expression has been reported to be upregulated predominantly in microglia in the SDH after traumatic nerve injury [20,21]. Thus, microglial cells could be a candidate for the source of CCL3 in the SDH of paclitaxel-treated rats. However, we can not exclude a possible involvemen t of other cell types expressing CCL3 in the SDH. Indeed, in addition to microglia, cultured astrocytes also express CCL3 [22]. Consistent with previous studies, we also found that paclitaxel treatment changed the morphology and expression of GFAP in the SDH (data not shown). Recent studies have implicated spinal astrocytes in chemotherapy-induced mechanical hypersensitivity [23,24].

The present study showed that a single intrathecal administration of either a CCL3-neutralizing antibody or the selective P2X7R antagonist reversed the established mechanical allodynia in paclitaxel-treated rats. Considering that microglia release CCL3 in response to P2X7R activation [11], the most parsimonious hypothesis is that ongoing signaling via CCL3 (presumably released from P2X7R-stimulating microglia) is crucial for maintaining paclitaxel-induced pain hypersensitivity. Although the detailed mechanisms by which CCL3 mediates paclitaxel-induced mechanical allodynia are not clear, spinal CCL3 might affect spinal pain processing. The ability of spinal CCL3 to produce mechanical allodynia has been demonstrated by our and other studies showing that intrathecal administration of CCL3 to naïve animals produces pain hypersensitivity in response to mechanical stimulation [12,25]. The expression of CCR5 in the SDH was markedly upregulated, and the time course of its upregulation matched that of CCL3 expression in the SDH. CCR5 was implicated in pain hypersensitivity in a model of neuropathic pain caused by traumatic nerve injury [12,15]. CCR5 expression has been reported to be localized to activated microgila in the spinal cord in response to nerve injury [12,26]. It is thus speculated that spinal CCL3 released from microglia in response to activation of P2X7Rs by extracellular ATP (which is presumably released from neighboring neurons [27] or glial cells [28]) further activates microglia via CCR5 in an autocrine manner, which may in turn lead to an alteration of dorsal horn pain processing. On the other hand, there was a lack of changes in CCR1 expression in the SDH of pactitaxel-treated rats, although a previous study has reported the upregulation of CCR1 in the spinal cord after traumatic nerve injury [13]. Considering recent findings showing the failure of a selective CCR1 antagonist to suppress paclitaxel-evoked cold hypersensitivity [29], spinal CCL3-CCR5 signaling may play an important role in paclitaxel-induced neuropathic pain. However, how CCL3-stimulated microglia affect pain processing in the dorsal horn remains to be determined.

In summary, the present study revealed CCL3 as an important player in the development and maintenance of mechanical allodynia following paclitaxel treatment. Therefore, our findings not only provide evidence for a new mechanism underlying the pathogenesis of chemotherapy-induced peripheral sensory neuropathy, but also suggest a novel therapeutic approach to neuropathic pain.

## Competing interests

The authors declare that they have no competing interests.

## Authors’ contributions

RO designed, performed the experiments, analyzed the data, and wrote the manuscript; KN and TI designed and performed the experiments; HS-T analyzed the data; MT supervised the overall project, and wrote the manuscript. KI conceived the study, supervised the overall project, and wrote the manuscript. All authors discussed the results and commented on the manuscript. All authors read and approved the final manuscript.
